# Takotsubo cardiomyopathy complicated by cardiac tamponade due to non-hemorrhagic pericardial effusion: a case report

**DOI:** 10.1186/s12872-020-01377-5

**Published:** 2020-02-06

**Authors:** Yuta Nagamori, Takuto Hamaoka, Hisayoshi Murai, Shinichiro Takashima, Takeshi Kato, Soichiro Usui, Kenji Sakata, Hiroshi Furusho, Masaaki Kawashiri, Masayuki Takamura

**Affiliations:** Department of Cardiology, Graduate School of Medical Science, Kanazawa University, 13-1 Takara-machi, Kanazawa, 920-8641 Japan

**Keywords:** Takotsubo cardiomyopathy, Pericarditis, Cardiac tamponade

## Abstract

**Background:**

Cardiac tamponade is a rare but serious complication of Takotsubo cardiomyopathy (TC). Two cases of cardiac tamponade subsequent to TC have been reported. The pericardial effusion in these cases was hemorrhagic and caused by ventricular rupture. Cardiac tamponade induced by an inflammatory effusion complicated with TC has not been reported. This is the first case report of TC, which developed cardiac tamponade during the recovery phase with a large volume non-hemorrhagic inflammatory effusion.

**Case presentation:**

We describe a case of an 81-year-old woman admitted to our hospital because of severe chest pain. Her symptoms began soon after her son’s hospitalization. We diagnosed her with TC based on results of an electrocardiogram, echocardiogram, and emergent coronary angiography. Her symptoms and left ventricular dysfunction improved gradually. She developed newly confirmed chest pain and dyspnea on day 9 after admission. A large pericardial effusion developed, resulting in cardiac tamponade. Her symptoms and hemodynamic status improved immediately after the pericardiocentesis. The effusion was non-hemorrhagic and exudative. No specific signs of infection, collagen disease, or malignant tumors were observed, except for TC.

**Conclusions:**

We experienced a case of circulatory collapse induced by TC-related inflammatory pericardial effusion at recovery phase. This case emphasizes the importance of careful follow-up even after improved left ventricular dysfunction in a patient with TC.

## Background

Takotsubo cardiomyopathy (TC) is characterized by transient dysfunction of the left ventricular mid-apical segments without significant coronary artery stenosis or occlusion that is generally induced by emotional or physical stress. TC always has a benign course; however, serious complications can develop, including cardiogenic shock, dysrhythmia, or ventricular rupture. Pericardial effusion is another well-known complication of TC, but it rarely affects the hemodynamic status. Two cases of cardiac tamponade subsequent to TC have been reported [1, 2]. The pericardial effusion in these cases was hemorrhagic and caused by ventricular rupture. Cardiac tamponade induced by an inflammatory effusion complicated with TC has not been reported. Here, we report a patient with TC who developed cardiac tamponade during the recovery phase with a large volume non-hemorrhagic inflammatory effusion.

## Case presentation

An 81-year-old woman presented to our hospital with severe chest pain for the prior 3 days. She had no medical history. She had never smoked and had no family history of cardiovascular disease. The symptoms began soon after her son was admitted to the hospital.

**Fig. 1:**
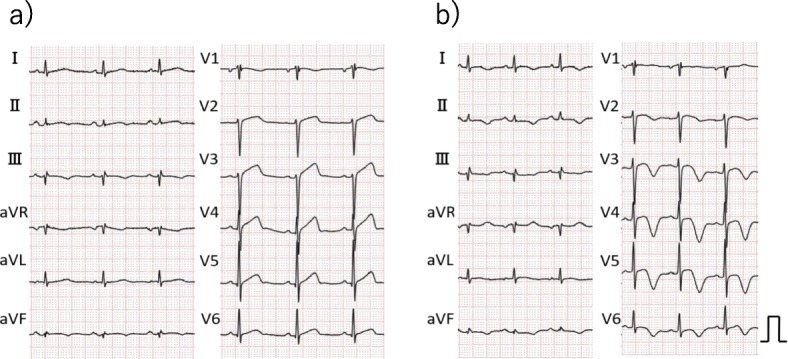
ECG (**a**: at presentation **b**: on day 9). **a** ST-segment was elevated in leads V2 to V5, III, and aVF. **b** T waves were inverted in the leads where the ST segment had been elevated previously

**Fig. 2:**
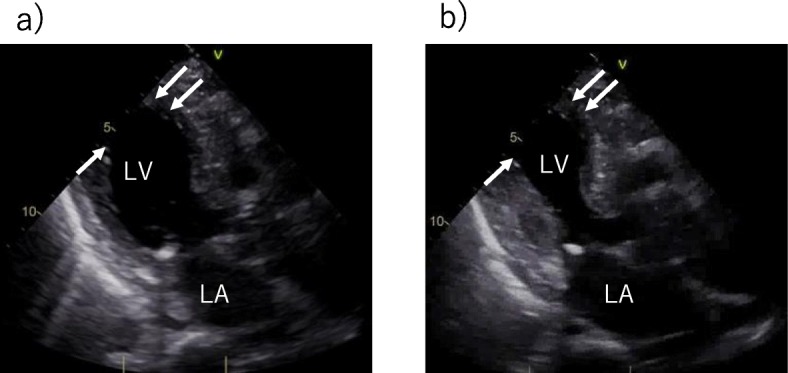
Echocardiography at presentation (**a**: diastole **b**: systole). The apical region of the left ventricle was akinetic (white arrows), and there was no pericardial effusion.(LA, Left Artium; LV, Left Ventricle)

**Fig. 3:**
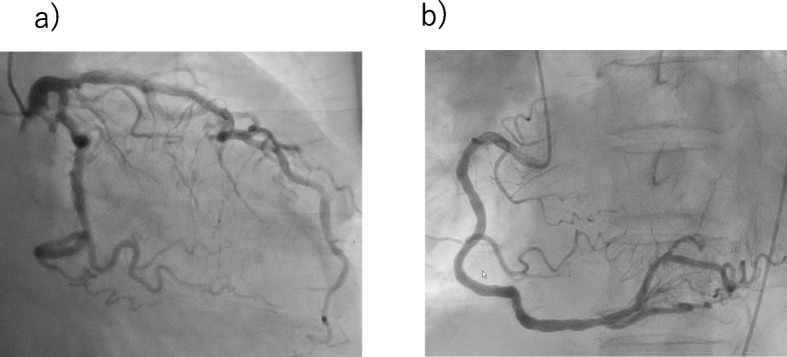
Coronary angiography. **a** Right anterior oblique 30°**b** Left anterior oblique 45°. No significant coronary artery stenosis or occlusion was detected

**Fig. 4:**
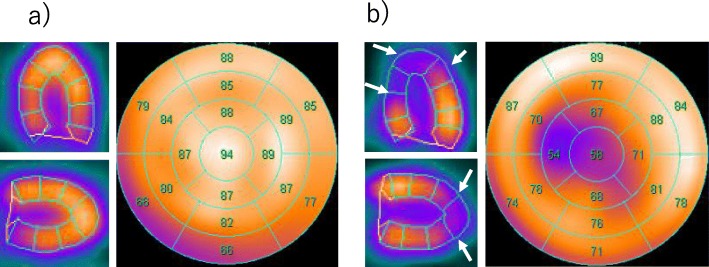
Nuclear cardiology examination (**a**: ^201^Thallium **b**: ^123^I-β-methyl-p-iodophenyl-pentadecanoic acid). Metabolic blood flow mismatches were detected in the left ventricular apical region (white arrows)

The initial electrocardiogram (ECG) revealed a normal sinus rhythm with ST-segment elevation in leads V2 to V5, III, and aVF (Fig. 1-a). A chest X-ray showed no pulmonary congestion or pleural effusion. Echocardiography revealed akinesis in the left ventricular apical region with hypercontraction in the basal region. No pericardial effusion was observed (Fig. 2). Laboratory studies demonstrated a small elevation in cardiac enzymes: creatine kinase, 125 IU/l (normal, 32–170 IU/l); creatine kinase-MB isoenzyme, 25 IU/l (< 16 IU/l); troponin T, 0.026 ng/mL. Neither neutrophils nor C-reactive protein were elevated (~ 0.03 mg/dl). Coronary angiography was performed, but no significant coronary artery stenosis or occlusion was detected (Fig. 3). Metabolic blood flow mismatches were detected in the left ventricular apical region on a nuclear cardiology examination (Fig. 4); these were inconsistent with the coronary artery perfusion area. From these results, this patient was diagnosed with Takotsubo cardiomyopathy (TC). Her symptoms had completely improved by 3 days after admission, and cardiac rehabilitation with a careful follow-up proceeded.

**Fig. 5:**
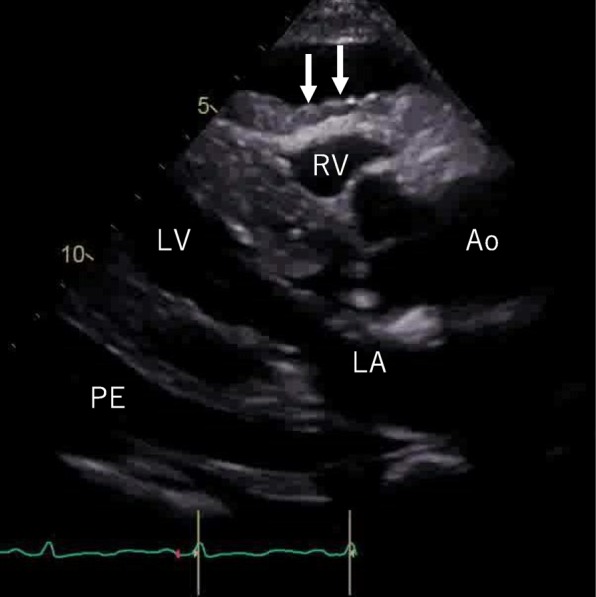
Echocardiography on day 9 (diastole). Pericardial effusion up to 10 mm appeared behind the left ventricle inferior-lateral lesion, leading to the collapse of the right ventricle (white arrows) (LA, Left Atrium; LV, Left Ventricle; RV, Right Ventricle; Ao, Aorta; PE, Pericardial Effusion)

**Fig. 6:**
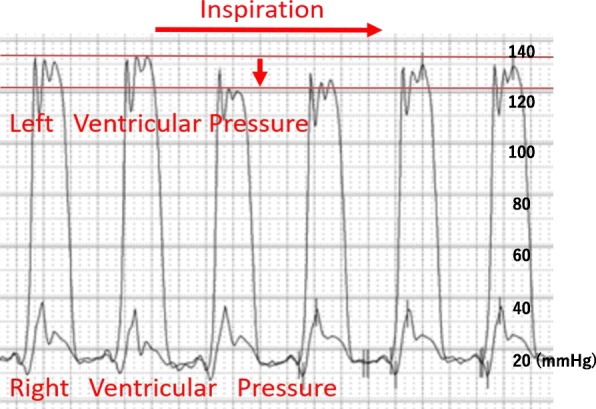
Cardiac catheterization. Average intracardiac diastolic pressures was equilibrated, and systolic blood pressure was reduced more than 20 mmHg by inspiration

**Fig. 7:**
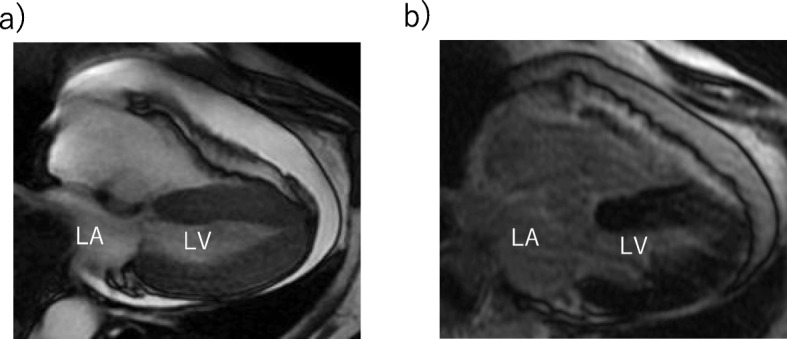
Magnetic resonance imaging (**a**: Fat suppressed T2 weighted image **b**: Delayed gadolinium enhancement image). Neither transmural late gadolinium enhancement nor cardiac edema were observed. (LA, Left Atrium; LV, Left Ventricle)

On day 9 of hospitalization, she felt mild chest pain at rest, and severe exertional dyspnea. An ECG revealed inverted T-waves in the leads where the ST segment had been elevated previously (Fig. 1-b). The left ventricular dysfunction had improved on echocardiography, but pericardial effusion of up to 10 mm appeared behind the left ventricle inferior-lateral region, indicating pericarditis. Loxoprofen 75 mg and colchicine 0.5 mg per day were administered, but the pericardial effusion increased gradually, leading to collapse of the right ventricle with sinus tachycardia (Fig. 5). A paradoxical pulse was also detected, with systolic blood pressure dropping to 21 mmHg during the inspiration phase. Cardiac catheterization revealed equilibration of the average intracardiac diastolic pressure between the left and right ventricles, and > 20 mmHg systolic blood pressure reduction by inspiration; these results were consistent with cardiac tamponade (Fig. 6). Pericardiocentesis was performed on day 23. The pericardial effusion was non-hemorrhagic and exudative. Malignant tumors, collagen disease, and tuberculosis were not thought to have introduced the pericardial effusion (WBC 0.45, TP 5.0 IU-L, LDH 291 IU/L, ADA 9.0 U/L, CEA 0.3, SCC 0.6, NSE 2.7 ng/mL, CYFRA 10.8, antinuclear antibody 80, cytodiagnosis negative). The serum virus paired antibody test was negative for echovirus and coxsackie virus. Therefore, only TC was a suspected cause of the pericarditis. On day 30, cardiac magnetic resonance imaging (CMRI) was performed to evaluate myocardial involvement, and no delayed gadolinium enhancement (DGE) was observed (Fig. 7). After draining 480 ml of fluid, the patient’s symptoms and abnormal hemodynamic status improved immediately. No additional pericardial effusion or relapse of symptoms has been observed.

## Discussion and conclusions

The present case exemplifies two important clinical issues. First, TC can induce pericarditis even during the recovery phase. Second, TC-related inflammatory effusion is capable of disrupting hemodynamic status.

### Relationship between Takotsubo cardiomyopathy and pericarditis

Some cases of pericarditis complicated with TC have been reported; however, the mechanism remains unclear. Two hypotheses for the relationship have been suggested. One is that pericarditis precedes the onset of TC, and intense sympathetic stimulation due to severe pericarditis-related chest discomfort subsequently induces the TC [3]. The other hypothesis is that myocardial inflammation of TC spreads to the pericardium, resulting in pericarditis [4]. In the present case, echocardiography initially demonstrated left ventricular dysfunction without pericardial effusion, and the pericardial effusion developed after amelioration of left ventricular function. The results of coronary angiography, a nuclear cardiology examination, and anamnesis indicated that the left ventricular dysfunction in the present case was derived from TC. The effusion sample was exudative; however, no remarkable signs suggesting malignant diseases, infection, or a collagen disease were observed. We speculate that the TC developed first, and the myocardium inflammation from TC induced pericarditis, which supports the hypothesis that TC precedes pericarditis. It can be difficult to differentiate TC from a coronary artery multi-vessel spasm or myocarditis [5]. In the present case, the cardiac enzymes were too low to be indicative of ischemic heart disease involving multiple vessels. In addition, CMRI demonstrated no region of DGE. A previous study showed that typical DGE is usually observed in ischemic heart disease and myocarditis; however, it is not observed in 95% of patients with TC [6]. These results confirmed our diagnosis of TC and TC-related pericarditis.

### Severity of pericarditis complicated with TC

Pericardial effusion complicated with TC is not rare. Ingo et al. reported that 43% of patients with TC develop pericardial effusion, and 81% of patients with TC have demonstrated myocardial edema on CMRI [7], but most of these cases were asymptomatic. Some symptomatic pericarditis cases complicated with TC have been reported [8]. In most of these cases, non-steroidal anti-inflammatory agents were effective, and no additional treatment was needed. Two cases, in which pericardiocentesis was applied to improve the cardiac tamponade, have been reported [1, 2]. The pericardial effusion in these cases was hemorrhagic, derived from cardiac rupture, and no studies on cardiac tamponade complicated with TC-related inflammatory effusion have been reported. In the present case, the pericardial effusion increased rapidly, resulting in cardiac tamponade regardless of the use of anti-inflammatory agents, including non-steroidal anti-inflammatory agents and colchicine. The effusion sample was exudative and non-hemorrhagic, indicating pericardial inflammation. After the pericardiocentesis, no additional effusion pooled, suggesting improvement in the pericardial inflammation.

As described above, TC-related symptomatic pericarditis is uncommon; however, in the present case and a previous report, pericarditis developed during the TC recovery phase [4]; thus, if a detailed examination is delayed, it is difficult to differentiate pericarditis related to TC from that from other origins. Cases of pericarditis with an identified etiology include malignant diseases (5–10%) systematic inflammatory disease and pericardial injury syndrome (5–10%), tuberculosis (4%), and purulent pericarditis (< 1%). About 80–90% of cases are labeled idiopathic, and most are presumed to be viral [9]. Some TC-related cardiomyopathy might be concealed in these cases of idiopathic pericarditis.

The present case had some limitations. First, although pericarditis was indicated, no systemic inflammation was observed. This can be explained by the results of a previous study, which demonstrated fewer systemic inflammation signs in patients with pericarditis with myocardial involvement than in those in simple pericarditis [10]. Second, some reports have suggested specific signs in TC evaluated by CMRI (cardiac edema evaluated by T2 weighted image) [7], but CMRI used in the present case revealed no remarkable signs. The appearance of signs with this technique is likely to be affected by the timing of the measurement. Myocardial edema has been observed in TC during the subacute phase, but not during the acute or chronic phases [11]. In the present case, CMRI was performed 30 days after admission. We might have detected the myocardium edema if we had performed CMRI at the subacute phase. In conclusions, most TC-related pericardial effusion is asymptomatic; however, it is capable of affecting hemodynamics. The condition of these patients should be carefully evaluated, even after improvement of left ventricular function.

## Data Availability

Not applicable.

## Abbreviations

CMRI: Cardiac magnetic resonance imaging
DGE: Delayed gadolinium enhancement
ECG: Electrocardiogram
TC: Takotsubo cardiomyopathy
